# Maternal and cross-stage effects of *Metarhizium pingshaense* fungal infection on the malaria mosquito *Anopheles coluzzii* life-history

**DOI:** 10.1371/journal.pone.0355106

**Published:** 2026-08-10

**Authors:** Issiaka Sare, Mafalda Viana, Souro Abel Millogo, Doubé Lucien Lamy, Assita Tenetao, Athanase Badolo, Florencia Djigma, Abdoulaye Diabate, Francesco Baldini, Etienne Bilgo

**Affiliations:** 1 Institut de Recherche en Sciences de la Santé, Direction Régionale de l’Ouest, Bobo-Dioulasso, Burkina Faso; 2 Institut National de Santé Publique/ Centre Muraz, Bobo Dioulasso, Burkina Faso; 3 Laboratoire d’Entomologie Fondamentale et Appliquée (LEFA), Université Joseph Ki-Zerbo, Ouagadougou, Burkina Faso; 4 Laboratoire de Biologie Moléculaire et de Génétique (LABIOGENE), Ecole Doctorale Sciences et Technologie, Université Joseph Ki-Zerbo, Centre de Recherche Biomoléculaire Piétro Annigoni (CERBA), Ouagadougou, Burkina Faso; 5 School of Biodiversity One Health and Veterinary Medicine, University of Glasgow, Glasgow, United Kingdom; 6 Université Nazi Boni, Bobo Dioulasso, Burkina Faso; 7 Environmental Health, and Ecological Sciences Department, Ifakara Health Institute, Morogoro, United Republic of Tanzania; Ladoke Akintola University of Technology, NIGERIA

## Abstract

**Background:**

Entomopathogenic fungi of the genus *Metarhizium* are widely used as eco-friendly biocontrol agents against insect vectors, offering a cost-effective alternative to chemical insecticides. While these fungi have demonstrated potential as larvicides against malaria vectors, their impacts on mosquito life-history traits and maternal effects remain poorly understood. This study evaluated the effects of locally isolated *Metarhizium pingshaense* strains (S10 and S26) on survival, development, reproductive fitness, and maternal effects in *Anopheles coluzzii* mosquitoes.

**Methods:**

We assessed the efficacy of *M. pingshaense* (strains S10 and S26) against both larval and adult stages of *An. coluzzii*. For larval assays, third-instar larvae were reared in water containing 2 × 10^5^ conidia/mL of *M. pingshaense*. Adult survival, wing length, oviposition rate, and blood-feeding behavior were subsequently measured. For adult assays, female mosquitoes were sprayed with spore suspensions of strains S10 and S26, and survival of their offspring was assessed. Survival data were analyzed using a Cox proportional hazards model, while other life-history traits were analyzed using generalized linear mixed models (GLMMs). All experiments were performed in triplicate to ensure reproducibility.

**Results:**

Exposure to fungal suspensions during the larval stage caused significant mortality at the pupal stage (~30%) compared to untreated controls. Adults emerging from treated larvae showed reduced survival (~23%) relative to controls, while wing length and blood-feeding behavior were unaffected. Adult female exposure to fungal spores increased egg-laying compared to controls, but a lower proportion of those larvae developed successfully into adults, demonstrating cross-stage and maternal effects.

**Conclusion:**

The results demonstrate the potential of *M. pingshaense* conidia as a biocontrol agent for *An. coluzzii* larvae, with additional cross-stage and maternal effects that further reduce mosquito fitness. These findings support the inclusion of *M. pingshaense* in integrated vector management strategies. Future work should focus on elucidating the molecular mechanisms underlying larval susceptibility and optimizing fungal formulations to enhance virulence.

## Introduction

The widespread use of insecticide-treated nets at the turn of the century was associated with a marked reduction in malaria incidence and mortality [1]. However, progress in reducing malaria cases has stalled in recent years, particularly in sub-Saharan Africa, with over 200 million cases reported annually and an estimated 400,000 deaths, imposing a substantial economic burden on endemic countries [2,3]. The increasing spread of insecticide resistance in malaria vector populations is a major factor contributing to the slowdown of control efforts [4,5]. Indeed, most vector control programs still rely heavily on chemical interventions such as long-lasting insecticidal nets (LLINs) and indoor residual spraying (IRS), highlighting the urgent need for complementary tools that are environmentally sustainable and effective against resistant mosquito populations [6]. One promising complementary approach is the use of larvicides or interventions targeting mosquito larvae. Traditional chemical larvicides, while effective, pose ecological risks due to toxicity to non-target organisms, persistence in the environment, and potential to drive resistance [7]. This has motivated the search for selective, biodegradable, and environmentally friendly biocontrol solutions. Among these, entomopathogenic fungi (EPF) have emerged as a highly promising class of bio-insecticides with narrow host specificity, minimal environmental impact, and demonstrated efficacy against major mosquito vectors [8].

EPF species such as *Metarhizium anisopliae* and *Beauveria bassiana* have shown specificity in infecting larvae and adults of mosquito genera including *Anopheles*, *Aedes*, and *Culex* [9]. Their mode of action involves the adhesion of conidiospores to the insect cuticle, germination, formation of appressoria, penetration into the hemocoel, proliferation within host tissues, and disruption of physiological processes, leading to systemic infection and death [10,11]. Fungal virulence is mediated by secondary metabolites that interfere with host immune defenses, energy reserves, and development, and can enhance susceptibility to additional stressors such as predation or chemical exposure [12,13].Experimental studies have demonstrated substantial efficacy of EPF in both laboratory and semi-field settings, including significant mortality in adult mosquitoes and potential larvicidal activity [14,15]. *Metarhizium* spores can persist in aquatic habitats, facilitating contact or ingestion by larvae, thereby disrupting development and reducing survival. Histopathological analyses have confirmed that EPF infections impair cuticle integrity, alimentary and respiratory systems, and metabolic functions, contributing to systemic mycosis and mortality [16,17]. Certain isolates of *B. bassiana* have also shown effectiveness against culicine mosquito larvae (*Culex* spp.), highlighting a broader spectrum of action [18].

Beyond direct lethality, EPF produce sublethal effects that can further suppress mosquito populations, including developmental delays, reduced pupation rates, compromised adult emergence, and altered reproductive capacity [18,19]. These multi-faceted effects position EPF as promising alternatives to conventional larvicides, particularly in the context of widespread insecticide resistance and the need for environmentally sustainable vector control strategies [20]. Previous studies by our group have shown that *M. pingshaense* strains S10 and S26**,** locally isolated in Burkina Faso, reduce adult mosquito survival**.** However, the effects of these strains on larval stages and the subsequent consequences for adult fitness, reproductive success, and potential cross-stage impacts remain poorly understood. The aims of the present study were to evaluate the efficacy of *M. pingshaense* strains S10 and S26 in controlling larval survival and adult emergence, and to assess the impact of fungal infection on life-history traits including wing size, feeding behavior, fecundity, and maternal effects. Cross-stage effects refer to the influence of infection or stress at one life stage on outcomes at another stage. In mosquitoes, maternal infection may affect offspring viability, development, or susceptibility to stressors. While such effects are poorly documented for EPF, understanding them is critical for predicting population-level impacts of biocontrol interventions. In this study, we specifically investigated whether larval infection with *M. pingshaense* affects subsequent adult fitness and reproductive traits, and whether maternal exposure influences offspring survival and development.

## Methodology

**Fig 1:**
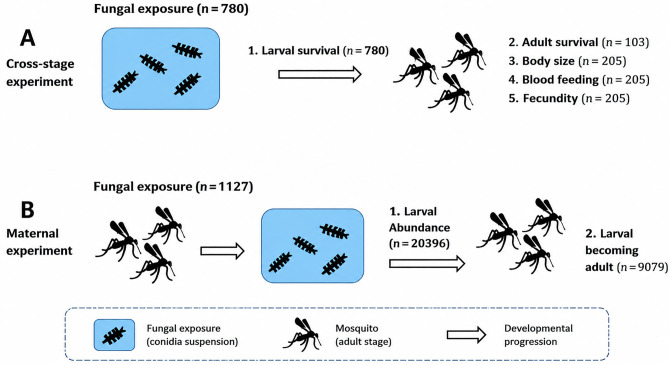
A. Assessment of fungal infection effects across mosquito developmental stages; B: Evaluation of maternal fungal infection impact on offspring traits.

We conducted two separate experiments to evaluate the effects of *M. pingshaense* infection across mosquito life stages. In the first experiment (Fig 1A). we assessed cross-stage effects by infecting mosquito larvae with *M. pingshaense*. In the second experiment (Fig 1B), we exposed unmated adult males and females to a *M. pingshaense* spore suspension and subsequently monitored life-history traits in their offspring. For this adult infection bioassay, female mosquitoes were first cold-anesthetized and transferred to a Petri dish. They were then sprayed with a fungal suspension at a concentration of 10⁷ spores/mL under a laminar flow hood. After exposure, the mosquitoes were placed in cardboard cups and maintained under standard insectary conditions. Mortality was recorded daily, and dead individuals were removed from the cups.

### Fungal strain origin

The fungal strains used in this study were obtained from the IRSS/Centre Muraz laboratory.

These strains are maintained under laboratory conditions and are identified as S10 and S26 according to their accession numbers in the strain library. Prior to experimentation, fungal isolates were cultured on Potato Dextrose Agar (PDA) and maintained at 27 ± 2°C

### Mosquito rearing

*Anopheles coluzzii* mosquito strain used in the experiment was from the 11^th^ generation of a line that originated from the Vallée du Kou and was established in the laboratory. The colony was maintained at 27 ± 2°C, relative humidity of 70 ± 5% and photoperiod of 12L:12D. This colony is known to have almost fixed L995F Kdr allele [21]. The larvae were kept in trays filled with tap water and fed at all stages with Tetra-min® (manufactured by tetra GMBH, HRERENTEICH 78 D-49324). All emerged adult mosquitoes were provided with continuous access to a 5% glucose solution throughout the experiment to ensure adequate nutrition, maintain physiological condition, and minimize any potential effects of nutritional stress on survival and life-history traits.

### Fungal suspension preparation

Using a sterile spatula, small fragments of fungal mycelium and conidia were carefully collected by gently scraping the surface of the fungal culture in a Petri dish under aseptic conditions to prevent contamination [22]. The harvested material was then suspended in a sterile aqueous solution containing 0.05% Tween 80, a non-ionic surfactant commonly used to improve conidia dispersion and prevent clumping. This suspension was vortexed for several minutes to ensure a homogeneous distribution of conidia.

To determine the conidia concentration, an aliquot of the suspension was subjected to quantification using a Neubauer hemocytometer under a phase-contrast microscope. The desired concentration of 107 conidia/mL was achieved by serial dilution or concentration adjustment, ensuring a standardized inoculum density for bioassays.

Once prepared, the conidial suspension was introduced into larval breeding trays containing mosquito larvae at stage 4 (L4). The trays were maintained under controlled environmental conditions, including temperature (27 ± 2°C), relative humidity (75 ± 5%), and a 12:12 h light-dark photoperiod, to mimic natural breeding habitats. The exposure period was standardized to allow sufficient fungal attachment and germination on larval cuticles, facilitating infection. This experimental setup enabled the assessment of fungal pathogenicity and virulence against mosquito larvae under laboratory conditions.

### Larval survival after fungal infection

A total of ten plastic trays (30 × 20.5 × 6.5 cm) for S10 and S26 and six trays for the control were used in the experiment, with each tray containing 30 L4 larvae. This setup was replicated three times, resulting in a total of 780 larvae. The larvae were exposed to the fungal solution and remained in the treated environment until they reached the pupal stage. Each tray contained a total volume of 50 mL, composed of 49 mL of tap water and 1 mL of a fungal suspension at an initial concentration of 10^7^conidia/mL. The addition of the fungal solution to the water led to a final concentration of 2.105 conidia/mL in each tray and we evaluated fungal effect on larval survival and on its metamorphosis to pupae stage. Larvae were fed on Tetra-min baby fish food. Then, we monitored and recorded larval survival rates throughout their development, from the larval stage to adulthood. For this experiments, three (03) replicates were carried out to evaluate fungal infection on larval survival.

### Survival of emerging adults

The adult mosquitoes that successfully emerged from the treated larval suspension were carefully collected and transferred to designated rearing cages (~ 30 mosquitoes per cage per treatment (S10, S26 and control)) under controlled conditions. Inside the cages, they were provided with a continuous supply of a 5% glucose solution to ensure proper feeding. Mortality was monitored daily, with dead mosquitoes being systematically removed from the cages for up to five days post-emergence. This allowed for the assessment of delayed mortality effects potentially caused by fungal exposure during the larval stage.

### Mosquitoes wing size measurement after fungal infection

The left wings of adult male and female mosquitoes were carefully dissected using fine-tipped forceps and a sterile needle to ensure precision and minimize structural damage. Each excised wing was then mounted onto a microscope slide with a coverslip for detailed morphometric analysis, following the methodology described by Yeap et al. (2013) [23].

High-resolution digital images of the mounted wings were captured using a Nikon SMZ1500 stereomicroscope (Nikon, Japan) equipped with an integrated camera. The imaging process was conducted at a magnification of 11.25X to ensure accurate measurement of wing dimensions. Wing length was determined by measuring the linear distance from the distal wing tip to the alular notch, a standard landmark for wing morphometry in mosquitoes. A total of 225 mosquitoes were analyzed for this experiment, with the study conducted in three independents replicates to ensure statistical robustness and reproducibility of the findings at three different times.

### Fungus infection effect on female mosquito fecundity and fertility

Four experimental cages, each containing approximately 100 mosquitoes (50 males vs 50 females) for each group, were used to assess the impact of two fungal strains (S10 and S26) exposure on mosquito reproductive success. The mosquitoes, aged 3–5 days post-emergence, originated from either the fungal-treated larval suspension or the untreated control group. To ensure adequate blood intake for egg development, adult female mosquitoes were offered blood meals from restrained rabbits under approved institutional animal care and use protocols. All animal procedures complied with relevant ethical regulations and are reported in accordance with the ARRIVE 2.0 guidelines (https://arriveguidelines.org). So, all mosquitoes were offered a rabbit blood meal twice, thereby maximizing the likelihood of successful feeding. Following blood feeding, blood fed females were individually transferred to oviposition cups to facilitate precise monitoring of egg-laying behavior. The total number of eggs laid per female was recorded to evaluate fecundity. A total of 205 females were analyzed for this experiment.

To assess egg viability, the collected eggs derived from treated (S10 and S26) and untreated (control) mosquitoes were submerged in 50 mL of tap water, and their hatching success was systematically evaluated. This step allowed for the determination of potential carryover effects of fungal exposure on mosquito reproductive output and offspring development.

### Effect of fungal infection of adult mosquitoes on their offspring (G1)

For each of two fungal suspension treatment (S10 and S26), approximately 100 blood-fed female mosquitoes were exposed to fungal suspensions in 10 independent replicates. Prior to fungal application, mosquitoes were temporarily immobilized by chilling in a freezer at –4°C for 15 seconds, ensuring minimal stress while facilitating uniform exposure to fungal spores. Immobilized mosquitoes were then transferred onto three different petri dishs (replicate/pedri dish) lined with sterile filter paper to maintain aseptic conditions and prevent contamination during the spraying process with three treatments (S10, S26 and control). Mosquitoes were sprayed with a 1 mL fungal suspension containing *M. pingshaense* conidia at a standardized concentration of 107 spores/mL, formulated in 0.01% (v/v) aqueous Tween80. This formulation was applied using an Ami pulverisateur (Zhejiang, China), a precision spray device designed to ensure consistent and homogeneous deposition of conidia on mosquitoes. Control mosquitoes were sprayed with 1 mL of 0.01% (v/v) aqueous Tween 80 alone, serving as a negative control to account for any effects of the spraying procedure. After the infection, mosquitoes were transferred to holding cups (7 cm diameter × 9 cm height) and maintained under controlled environmental conditions: temperature of 25°C, relative humidity of 70 ± 10%, and a 12:12-hour light-dark cycle. To sustain physiological activity, mosquitoes were provided a 5% glucose solution soaked in a cotton ball.

Egg-laying behavior was monitored by isolating fed females in individual oviposition cups and recording the total number of eggs laid. The resulting eggs were then submerged in tap water under controlled conditions to allow hatching. The number of larvae that reached the fourth instar (L4) stage was recorded as an indicator of early developmental success. These L4 larvae were subsequently monitored until adult emergence, and the number of successfully emerged adults was documented to assess the impact of maternal fungal infection on offspring survival and development.

### Statistical analysis

**Fig 2:**
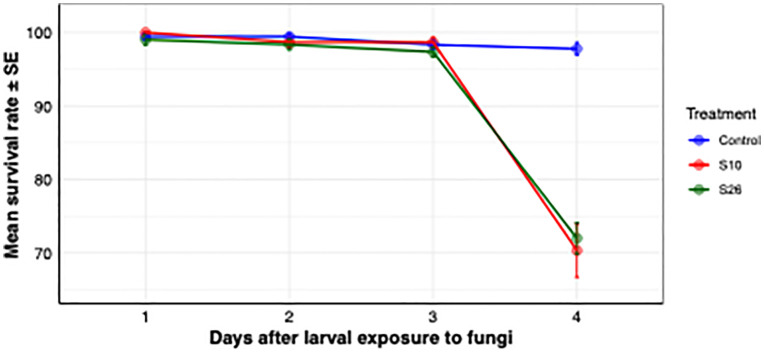
Cross-stage experiment, fungal effect on larval survival. Temporal changes in mosquito 4% instar larval survival under different treatments. Mean survival rates (* standard error) of 4t-instar larvae exposed to fungal isolates $10 (in red) and S26 (in green) compared to the untreated control (in blue) over a four-day period. Each point represents the average survival rate for a treatment group at a given time point. Lines connect points to show temporal trends, and error bars indicate standard error of the mean.

**Fig 3:**
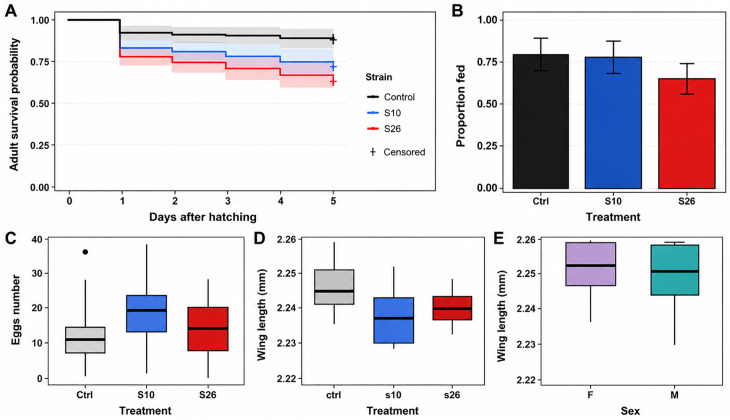
Cross-stage experiment: effects of fungal exposure during the larval stage on adult life-history traits. (A) Kaplan–Meier survival curves for adult mosquitoes emerging from larvae exposed to M. pingshaense strains S10 or S26, or the control treatment. Solid lines represent survival probabilities, shaded areas indicate 95% confidence intervals, and plus signs denote censored observations. (B) Proportion of females taking a blood meal. (C) Number of eggs laid per female. (D) Wing length according to treatment. (E) Wing length according to sex.

**Fig 4:**
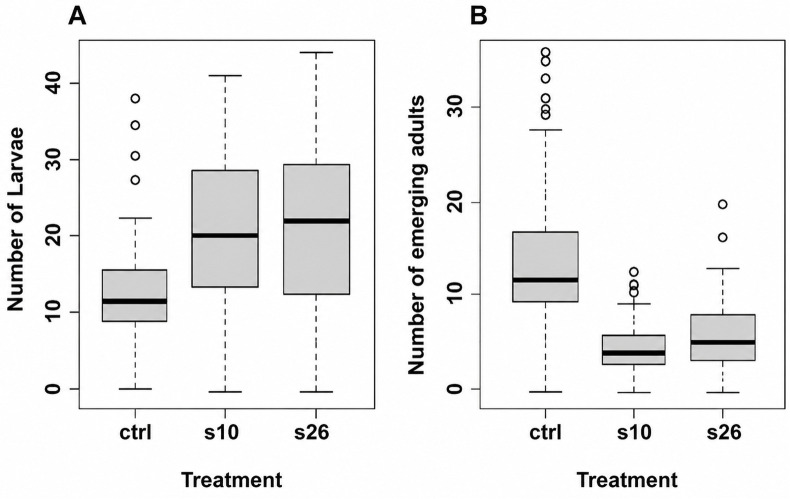
Maternal Experiment, fungal effect on progeny. Effect on adult fungal exposure on: A) number of L4 larvae hatched per female mosquito; B) Number of alive progeny adults per female mosquito.

Larval survival (Fig 2) rate was analyzed using a binomial Generalized Linear Model. Treatment (3 levels: Control, S10 and S26), time (4 levels: D1, D2, D3 and D4) and their interaction were included as fixed effects. A Cox proportional hazard models from the R package “survival”, was developed to determine the impact of fungal solution on the survival of adults (Fig 3A) [24]. For this, adult survival was the response variable while treatment was included as fixed effect and the random effect of ‘replicate’ was incorporated as a frailty function [25,26]. To understand the impact of the treatment on different entomological parameters of the adult mosquitoes, separate generalized linear mixed models (GLMMs) with negative binomial family distribution was developed with the following response variables:

proportion fed (Fig 3B); ii) number of eggs (Fig 3C); iii) Wing size (Fig 3 D&E), vi) hatched larvae (Fig 4A); v) generation G1(first offsprings from infected mothers) (Fig 4B) using the R package ‘glmmTMB’. Other parameters such as larval abundance were modelled using a negative binomial family distributed to account for the full dispersal in the data, while wing size was modelled following a Gaussian distributed GLMM with R package “lme4”. All these models were fitted with treatment as fixed effect and replicate as random effect. For all models described above, we performed model selection using stepwise removal of terms, followed by likelihood ratio tests. The best model retained only significant terms that improved model goodness. Model performance diagnostics (i.e., residuals and dispersion) were evaluated for all models using the R package ‘DHARma’. All statistical analyses were performed using R version 4.1.2.

### Ethical approval

Ethical permissions were obtained through the Institutional Review of Institut de Recherche en Science de la Santé (IRSS) ethic committee (N/Réf.A24-2020/CEIRES).

## Results

### Effect of two fungal strains S10 and S26 on 4^th^ instar larval survival

The result of larvae survival revealed that the two strains of *M. pingshaense* reduced larvae survival approximately around 30% at day 4 post exposure (survival rate with treated and controls: 70% vs 100%). There was a significant treatment × time interaction effect on larval survival rate (χ² = 17.79, df = 2, p = 0.006); At D1, D2 and D3 (days post exposure), L4 larval survival rate remained high (98–100%) in each treatment (S10, S26 and control); however, at D4, larval survival rate in the control treatment batch remained high (98–100%) while it was down to approx. 70% in both the S10 and S26 fungal treatment batches (Fig 2).

### Fungal exposure at larval stage reduced survival of emerging adults and increased their fecundity

Adult survival analysis showed a reduction in female survival 5 days after emergence in mosquitoes originating from fungal treatments compared to the control. Survival decreased by 12.5% in both *M. pingshaense* strains (S10 and S26), from 93.75% in the control to 81.25% in treated groups (χ² = 26.361, df = 2, p < 0.001) (Fig 3A). Blood-feeding rates were 78.26% in the S10 group, 65.43% in the S26 group, and 76.33% in the control group. Larval exposure to *M. pingshaense* did not significantly affect the proportion of females that successfully blood fed (χ² = 96.434, df = 2, p = 0.131) (Fig 3B. The proportion of female adults surviving fungal exposure that took a blood meal).

Females originating from fungal treatments laid more eggs than control females. A total of 1087 and 995 eggs were produced by S10 and S26-treated females, respectively, compared with 525 eggs in the control group. Overall, larval exposure to *M. pingshaense* increased egg production by approximately 1.27-fold relative to the control (χ² = 96.434, df = 2, p < 0.001) (Fig 3C).

### Larval fungal exposure did not alter the body size of emerged adults compared to control

Mosquito wing length, used as a proxy for body size, did not differ significantly between control and fungal treatment groups, indicating that larval exposure to *M. pingshaense* had no effect on adult mosquito size (χ² = 1.03, df = 2, p = 0.598) (Fig 3D). However, Females tended to be larger than males with a marginally significant difference (χ² = 3.90, df = 1, p = 0.048)(Fig 3E).

### Maternal fungal exposure increased the abundance of larval progeny but decreased the number of emerging adults compared to control

Maternal exposure to *M. pingshaense* significantly increased the number of L4 larvae produced compared with the control group. Larval abundance was 1.44-fold higher in the S26 treatment and 1.47-fold higher in the S10 treatment relative to the untreated control (χ² = 654.57, df = 2, p < 0.001). (χ² = 654.57, df = 2, p < 0.001) (Fig 4A). However, the number of larvae that became adults was two times lower in the groups treated with the fungi than in the untreated ones (χ² = 642.49, df = 2, p < 0.001) (Fig 4B).

## Discussion

Efficient management of mosquito breeding sites could provide a powerful complementary tool for malaria control. This may include the use of natural enemies such as entomopathogenic fungi, including *Metarhizium* [27,28]. Our results confirm previous findings that *Metarhizium* can effectively target malaria mosquitoes and provide additional insights into cross-stage effects in *Anopheles coluzzii* [29,30]. We observed that although fungal exposure reduced larval survival, individuals that reached adulthood also exhibited reduced survival. Interestingly, fecundity increased in surviving females, which laid eggs that appeared white in color, suggesting immature eggs. Together, these findings indicate trade-offs associated with *M. pingshaense* exposure across different mosquito life-history traits, including survival and reproduction. Our results suggest that fungal exposure at the larval or maternal stage can influence subsequent life stages in *An. coluzzii*, highlighting potential carry-over effects relevant for malaria vector control.

We found that infecting adult mosquitoes increased the number of larvae produced in the treated population compared to the control population. This suggests that infected individuals may have increased investment in early reproduction in response to reduced survival prospects, consistent with a terminal investment strategy. This finding is consistent with previous studies showing that entomopathogenic fungi, including *Beauveria bassiana* and *Metarhizium anisopliae*, can profoundly affect reproductive traits and offspring performance in insect hosts, although the direction and magnitude of these effects may vary among species and experimental conditions [13,31]. Their impact may extend beyond direct mortality, as fungal exposure can also influence key mosquito life-history traits such as survival and reproduction, which may ultimately affect mosquito population dynamics and thus their relevance for biological control strategies. However, we noted that a very small proportion of these larvae reached the adult stage in the next generation. Since the larval density was standardized across treatments, we believe the increased mortality cannot be explained by density pressure. Instead, a plausible explanation could be that the eggs were immature, so those that hatched did not possess the necessary biological components for proper development. The maternal effects observed in this study may be explained by pathogen-induced life-history trade-offs, whereby infected females reallocate resources from somatic maintenance and immune functions toward reproduction when future survival is compromised. Such terminal investment responses have been reported in several insect species exposed to pathogens and can result in increased reproductive effort despite reduced parental fitness [32]. In addition, fungal infection may alter maternal provisioning of eggs through effects on nutrient reserves, endocrine regulation, or immune activation, thereby reducing offspring quality and developmental success. These mechanisms could explain the higher larval production but lower adult emergence observed among the progeny of fungus-exposed females. Regarding larval exposure, our results suggest that this mode of application facilitates the dispersion of fungal spores across the water surface, thereby increasing the likelihood of contact with and infection of mosquito larvae. We found high mortality of the larvae in both treatments at the pupal stage. This suggests that the larvae ingested a lethal dose of the fungus as food during their larval stage, similar results have been reported [33,34]. These studies reported that entomopathogenic fungi infect insect hosts through the adhesion of conidia to the cuticle, followed by germination, penetration of the cuticle, and proliferation within the hemocoel, ultimately leading to host mortality. These studies also reported that fungal infection involves the production of virulence factors and secondary metabolites that disrupt host physiological processes and interact with the insect immune system, contributing to reduced survival and fitness.

Adults emerging from fungus-exposed larvae had a relatively low survival rate compared to control group. We hypothesize that the spore had already passed through the larval cuticle before moulting into the adult stage [35,36], so these spores expressed their toxins once in the adult mosquito’s haemolymph or that, even in the larval stage, the fungus had already passed through the mosquito’s haemolymph. Indeed, as *An. coluzzii* larvae have different rates of filtration and ingestion on the surface and the spore can infect by ingestion or contact [37]; However, for those larvae that survived the infection, it is possible that the spore was still on the surface of the host and that during molting the spore was shed or that there was no contact between the larva and the spore [16,38]. Molting has been reported to be an important factor in the resistance of arthropods to fungal infection, particularly in arthropods with short ecdysis intervals [39]. In a context where the multiplicity of mosquito breeding sites is a real bottleneck for malaria control programs [40,41]. The use of fungi in larval breeding sites could help to reduce mosquito populations at both the larval and adult stages [11,42]. Assessment of wing size and sex of treated and untreated individuals showed no significant difference. This is consistent with previous studies showing similar wing size measurements between untreated and treated individuals with the fungi, but they show females were generally larger than males, consistent with previous findings [23]. This suggests that infection at the larval stage does not influence the size of individuals according to their treatment status; however, females appear larger than males emerging from the fungal solution [43,44].

Based on the data collected regarding larval exposure to entomopathogenic fungi, the results can be interpreted as follows: infected larvae tend to occur at lower densities within their habitat, which may promote enhanced individual growth and, consequently, lead to higher egg production at the adult stage. However, this hypothesis is not supported by body size measurements, which did not reveal significant differences between infected and control individuals. Therefore, the increased fecundity observed in fungus-exposed females is unlikely to be explained by body size variation. Consequently, although egg production was higher, fewer offspring reached adulthood, highlighting a potential mechanism through which entomopathogenic fungi may contribute to mosquito population suppression [45,46]. While our results offer promising insights into the impact of *M. pingshaense* on multiple mosquito life-history traits, several limitations should be acknowledged. First, the experiments were conducted under laboratory conditions, which do not fully replicate the complexity of natural environments such as variable temperature, microbial communities, or habitat heterogeneity. Second, a limited number of fungal strains (2) were used, which may not capture the full spectrum of virulence, persistence, or environmental adaptation seen in wild populations. Third, our study did not include long-term or field-based data, making it difficult to predict the durability and population-level outcomes of fungal exposure under operational conditions. Future studies should incorporate semi-field and field trials, test a broader range of fungal isolates, and assess multi-generational effects under fluctuating environmental conditions to validate these findings and improve their translational potential

## Conclusion

*M. pingshaense* appears to be a promising biocontrol agent against *Anopheles coluzzii* mosquitoes. Our results indicate that, although infected adults laid more eggs, most were immature and few offspring reached adulthood, while larval exposure significantly reduced survival. These cross-stage effects suggest that *M. pingshaense* can influence mosquito population dynamics. Feeding behavior, oviposition, and adult body size were not significantly affected at the concentration tested, but adult lifespan was substantially reduced following fungal infection. Furthermore, fungal spores impacted larval survival and adult emergence, which could have important consequences for vectorial capacity. Taken together, these findings highlight the complex trade-offs induced by fungal infection and support the integration of entomopathogenic fungi into vector control strategies as a complementary tool to synthetic insecticides.

## Supporting information

S1 File Feeding. (CSV)

S2 File Generation. (CSV)

S3 File Larva_adult_survival-data. (XLSX)

S4 File Larvicidal-wing_feeding_data. (XLSX)

S5 File Wing_size. (CSV)

## Abbreviations

B: Beauveria
An: Anopheles
M: Metarhizium
Crtl: control group
S10: Metarhizium strain 10 in our stump library
S26: Metarhizium strain 26 in our stump library
